# Neurodevelopmental disorders among young adults and the risk of sickness absence and disability pension: a nationwide register linkage study

**DOI:** 10.5271/sjweh.3888

**Published:** 2020-07-01

**Authors:** Marianna Virtanen, Tea Lallukka, Mika Kivimäki, Kristina Alexanderson, Jenni Ervasti, Ellenor Mittendorfer-Rutz

**Affiliations:** Department of Clinical Neuroscience, Division of Insurance Medicine, Karolinska Institutet, Stockholm, Sweden; School of Educational Sciences and Psychology, University of Eastern Finland, Joensuu, Finland; Clinicum, Faculty of Medicine, University of Helsinki, Finland; Finnish Institute of Occupational Health, Helsinki, Finland; Department of Epidemiology and Public Health, University College London, UK

**Keywords:** Key terms ADHD, attention-deficit hyperactivity disorder, autism, learning disability, register study, sick leave

## Abstract

**Objectives:**

Attention-deficit/hyperactivity disorder (ADHD), autism spectrum disorders (ASD) and learning disabilities (LD) have an early onset and often persist into adulthood, although their relative contribution to incapacity for work is unclear. We examined this issue among young adults with ADHD, ASD or LD taking into account socioeconomic factors and comorbid mental disorders.

**Methods:**

Recorded diagnoses between the ages of 10–35 years between 2001 and 2010 were derived from nationwide inpatient and specialized outpatient hospital registers in Sweden. We identified 15 632 individuals with a main diagnosis of ADHD, 8238 with ASD, and 1038 with LD, and the matched control group without recorded mental disorders (N=124 536). The outcome was the number of register-based sickness absence and work disability pension (SA-DP) days during a maximum of three years follow-up.

**Results:**

Among men, the rate ratio (RR) of SA-DP was 11.17 [95% confidence interval (CI) 9.89–12.60] for ADHD, 35.59 (95% CI 30.30–41.81) for ASD, and 9.20 (95% CI 5.76–14.70) for LD, in comparison to those in the reference group. The corresponding risks among women were RR 12.05 (95% CI 10.30–14.09) for ADHD, RR 28.36 (95% CI 22.96–35.02) for ASD, and RR 9.60 (95% CI 5.83–15.81) for LD. The findings were, to a large extent, similar when individuals on DP at baseline were excluded. Comorbid mental disorders further increased the risk of SA-DP. Educational differences were smaller among the patients than in the reference group.

**Conclusions:**

Early-onset neurodevelopmental disorders, particularly with comorbidity, have a far-reaching impact on adult life in terms of SA and DP.

Attention-deficit/hyperactivity disorder (ADHD), autism spectrum disorders (ASD), and learning disabilities (LD) usually begin in childhood and may significantly impact the future lives of affected children. These disorders often persist into adulthood, may increase the risk of other mental disorders, and are associated with failure in developmental tasks such as gaining education and employment (1–10). The prevalence of ADHD, ASD, and LD among adults has been estimated to be around 4% (11), 1% (12), and 1–2.5% (13), respectively, although exact estimates for adults are seldom available. Economic costs in terms of special educational needs, healthcare services, and productivity loss in the labor market are substantial (1, 10, 14–17). Comorbid diseases are believed to be major drivers of healthcare utilization and cost (15–17).

The effects of early-onset neuropsychiatric and behavioral disorders may be particularly detrimental during young adulthood, since this period includes important developmental tasks, such as completing an education and entering into the labor market. To date, most studies have focused on ADHD, with the outcome usually being general ‘productivity loss’ measured as costs (1, 17) or occupational injuries (18). Two studies reported increased sickness absence (SA) days, assessed by self-reported days during the past month (15, 19). More short-term disability days but no difference between the likelihood of SA among workers with and without ADHD was reported in one study (16). However, these three studies did not examine other disorders than ADHD (15, 16, 19) or relied on self-reported outcome data (15, 19). We are not aware of any studies that have compared ADHD, ASD, and LD or examined whether socioeconomic factors and comorbid conditions are associated with the incapacity for work outcomes in these disorders.

Using register data covering the whole population of Sweden, we examined the longitudinal associations of ADHD, ASD, and LD with SA and disability pension (DP) among young adults, in comparison to those in a matched reference group without recorded mental disorders, and assessed the contribution of socioeconomic characteristics and comorbid mental disorders.

## Methods

### Participants and procedure

**Figure 1:**
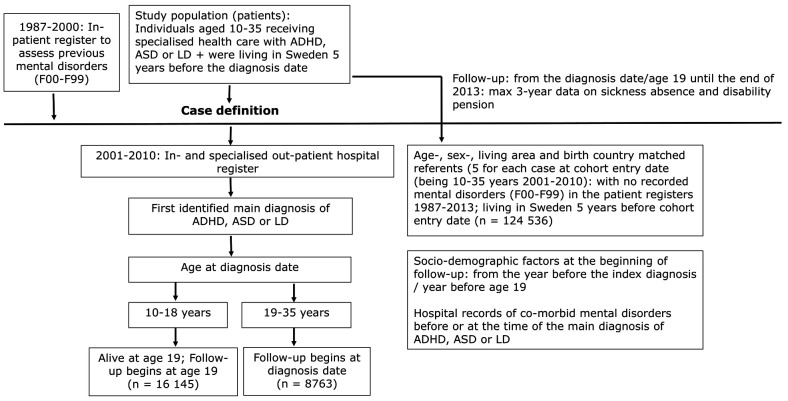
Study population. [ADHD=attention-deficit/hyperactivity disorder; ASD=autism spectrum disorders; LD=learning disabilities.]

The study population was a register-based cohort derived from the whole population’s register in Sweden, linked to in- and specialized outpatient registers between 2001 and 2010 (figure 1). The Regional Ethical Review Board, Stockholm, Sweden, approved the project. Statistics Sweden’s Longitudinal Integration Database for Health Insurance and Labor Market Studies (LISA) was used to obtain information on sex, age, education, birth country, and type of living area. The National Board of Health and Welfare provided data from the patient register (diagnosis-specific data on inpatient hospitalizations and specialized outpatient care), coded according to the International Classification of Diseases (ICD-10) and date of death. The National Social Insurance Agency provided information on annual days of SA-DP (the MIDAS register).

In Sweden, all individuals aged ≥16 years are entitled to SA benefits if they have income from work or unemployment benefits and have work incapacity due to disease or injury. All people aged 19–64, including those with no previous income, can be granted DP if their work capacity is long-term or permanently reduced due to disease or injury. For people aged ≥30 years, work incapacity has to be permanent before DP can be granted. Between ages 19–29, work incapacity needs to last for at least one year and can be granted to those still in school.

The individuals with neurodevelopmental disorders were those who had been 10–35 years old when their ADHD (International Classification of Diseases, ICD-10 code F90), ASD (F84.0, F84.1, F84.3, F84.5, F84.8, F84.9), or LD (F81) was recorded as the main diagnosis in the specialized in- or outpatient healthcare between 2001–2010. An additional inclusion criterion was living in Sweden five years prior to the treatment episode of specialized healthcare. Of the 24 908 individuals with a recorded diagnosis of ADHD, ASD, or LD, 65% were aged 10–18 at the time of their diagnosis in specialized healthcare, whereas 35% were aged 19–35 years.

We randomly selected five matched reference individuals (N=124 536) to each ADHD, ASD, and LD patient and matched them according to age, sex, type of living area, and birth country. The reference group had no indication of mental disorder (ICD-10 code F00-F99) in any of the hospital records. The individuals in the reference group had been living in Sweden five years prior to the cohort entry date of the patient with whom they were matched. A maximum of three years follow-up of annual net days of SA-DP began from the date of diagnosis, except when age at diagnosis was <19 years, in which case the follow-up began at the age of 19 (the eligibility age for DP in Sweden). The mean follow-up was 2.63 [standard deviation (SD) 0.69] years. The duration of follow-up depended on the year of entry into the cohort, death, and emigration. A total of 2593 individuals died or emigrated from Sweden during follow-up.

Covariates included sex, age, educational level (low: 0–9, intermediate: 10–12, high: ≥13 years), birth country (Sweden, other), type of living area (large city, medium sized town, small town/village), and comorbid mental disorders before or at the time of the main diagnosis, also derived from inpatient and specialized outpatient registers. Comorbidity also included mental retardation (ICD-10 codes F70-F73, F78, F79) (20). We used the MIDAS work DP register to identify those who were on work DP at the beginning of follow-up.

### Statistical analysis

Descriptive statistics (N, %) for each diagnostic group and the reference group were calculated for covariates. We calculated annual and 3-year net number of SA-DP days for each person (based on the sum of SA-DP). We applied negative binomial regression procedure to produce rate ratios (RR) and their 95% confidence intervals (CI) to estimate the difference in SA-DP between individuals with ADHD, ASD, or LD, and the reference group. The offset variable was logarithmically transformed duration of follow-up (maximum three years) calculated for all individuals. Models were adjusted for socioeconomic factors and the calendar year when follow-up began. The analyses were repeated among a sub-group of individuals who were not on DP at the beginning of follow-up. Further analyses were carried out separately among cases and the control group to examine the association between socioeconomic factors and SA-DP, and among cases, the association between comorbid mental disorders and SA-DP. We used SAS statistical software (9.4) (SAS Institute, Inc, Cary, NC, USA).

## Results

Table 1 presents the descriptive characteristics of individuals with ADHD, ASD and LD, and the reference group without recorded mental disorder. The largest diagnostic group was ADHD (N=15 632), followed by ASD (N=8238), and LD (N=1038). Men and those with low education were overrepresented in all the diagnostic groups. The highest prevalence of comorbid mental disorders was found among individuals with ASD (48%) and the majority of individuals with ASD (63%) were on DP already at the beginning of follow-up. The corresponding proportions for ADHD and LD were 22% and 21%, respectively.

**Table 1:** Characteristics at the beginning of follow-up of individuals with attention-deficit hyperactivity disorder (ADHD), autism spectrum disorder (ASD) and learning disabilities (LD), and a matched reference group without recorded mental disorders. [NA=not applicable; SD=standard deviation.]

	Reference group (N=124 536)		ADHD (N=15 632)		ASD (N=8238)		LD (N=1038)	
			
	Mean (SD)	N (%)	Mean (SD)	N (%)	Mean (SD)	N (%)	Mean (SD)	N (%)
Age at beginning of follow-up (reference)	21.2 (4.2)		21.2 (4.2)		21.3 (4.2)		20.0 (3.0)	
Sex								
Men		86 223 (69)		10 862 (69)		5755 (70)		628 (61)
Women		38 313 (31)		4770 (31)		2483 (30)		410 (40)
Type of living area								
Large city		41 619 (33)		5244 (34)		2793 (34)		287 (28)
Medium-sized town		45 565 (37)		5778 (37)		2910 (35)		425 (41)
Small town/village		37 352 (30)		4610 (29)		2535 (31)		326 (31)
Birth country								
Sweden		116 239 (93)		14 586 (93)		7701 (93)		961 (93)
Other		8297 (7)		1046 (7)		537 (7)		77 (7)
Educational level								
Low		21 741 (17)		10 065 (64)		5530 (67)		627 (60)
Medium		89 684 (72)		5041 (32)		2328 (28)		386 (37)
High		13 111 (11)		526 (3)		380 (5)		25 (2)
Comorbid mental disorder								
No		NA		9794 (63)		4264 (52)		753 (73)
Yes		NA		5838 (37)		3974 (48)		285 (27)
Disability pension at beginning of follow-up								
No		123 227 (99)		12 139 (78)		3029 (37)		825 (79)
Yes		1309 (1)		3493 (22)		5209 (63)		213 (21)

Multivariable-adjusted RR for SA-DP days during the 3-year follow-up among men and women are presented in table 2. Compared to the reference group, ASD was associated with a 35.6-fold risk of SA-DP among men and a 28.4-fold risk among women. ADHD was associated with an 11.2-fold risk among men and a 12.1-fold risk among women whereas LD was associated with 9.2-fold risk among men and 9.6-fold risk among women. The findings were largely similar among a sub-group consisting men and women not on DP at the beginning of follow-up although the absolute rates were remarkably lower.

**Table 2:** Rate ratio (RR) and 95% confidence intervals (CI) of sickness absence and disability pension days during follow-up among young adults diagnosed with attention-deficit/hyperactivity disorder (ADHD), autism spectrum disorder (ASD), or learning disabilities (LD) compared to matched reference group without recorded mental disorders.

	Among the total population (N=149 444)		Among a sub-group not on disability pension at beginning of follow-up (N=139 220)	
	
	Unadjusted rate per person-year	Adjusted RR (95% CI) ^a^	Unadjusted rate per person-year	Adjusted RR (95% CI) ^a^
Men				
Reference	5.1	1.00	2.2	1.00
ADHD	88.1	11.17 (9.89–12.60)	29.3	9.97 (8.29–11.99)
ASD	247.9	35.59 (30.30–41.81)	92.8	35.90 (25.54–50.46)
LD	70.8	9.20 (5.76–14.70)	18.5	6.60 (3.29–13.24)
Women				
Reference	6.1	1.00	3.3	1.00
ADHD	111.4	12.05 (10.30–14.09)	49.6	11.40 (9.10–14.29)
ASD	263.2	28.36 (22.96–35.02)	98.3	25.03 (16.22–38.65)
LD	78.4	9.60 (5.83–15.81)	19.2	6.01 (2.99–12.07)

a Adjusted for age, educational level, type of living area, birth country and year when follow-up began.

Among the patient groups, comorbid mental disorders further increased the SA-DP risk (table 3). The greatest RR (1.76) associated with comorbidity was found among those with ADHD. In ASD, the RR was 1.14 and in LD, the RR was 1.62 although not statistically significant in the latter group. Rather similar results were found among a sub-group of those not on DP at the beginning of follow-up.

**Table 3:** Rate ratio (RR) and 95% confidence interval (CI) of sickness absence and disability pension days during follow-up among young adults diagnosed with attention-deficit/hyperactivity disorder (ADHD), autism spectrum disorder (ASD), or learning disabilities (LD) by the presence of comorbid mental disorder

Diagnostic group and comorbidity	Among the total patient population (N=24 908)		Among a sub-group not on disability pension at beginning of follow-up (N=15 993)	
	
	Unadjusted rate per person-year	Adjusted RR (95% CI)^a^	Unadjusted rate per person-year	Adjusted RR (95% CI)^a^
ADHD				
No comorbidity	73.1	1.00	27.9	1.00
Comorbidity	132.3	1.76 (1.57–1.97)	50.3	1.60 (1.33–1.92)
ASD				
No comorbidity	233.8	1.00	81.4	1.00
Comorbidity	272.7	1.14 (1.06–1.23)	111.4	1.41 (1.09–1.82)
LD				
No comorbidity	61.1	1.00	15.2	1.00
Comorbidity	107.5	1.62 (0.90–2.92)	29.9	1.49 (0.42–5.26)

a Adjusted for age, sex, educational level, type of living area, birth country and year when follow-up began.

The associations between baseline socioeconomic characteristics and SA-DP are presented in table 4. In all groups, women had higher risk than men. There was much greater differences between educational groups among the reference group without mental disorders (RR for low versus high education 18.40) than among the patients (RR 2.98). However, the estimate for low education in the reference group diluted to RR 4.02 when the individuals on DP at baseline were excluded from the analysis (the corresponding RR 2.10 among the patient group). Living in a small town or rural area was rather consistently associated with higher risk of SA-DP when compared to living in a big city. Non-Swedish birth country was associated with lower risk of disability among the reference group without mental disorders, while no differences were observed in the patient group.

**Table 4:** Baseline characteristics associated with the rate ratio (RR) and 95% confidence interval (CI) of sickness absence and disability pension days during follow-up among young adults with attention-deficit/hyperactivity disorder (ADHD), autism spectrum disorder (ASD), or learning disabilities (LD) and among a reference group without recorded mental disorders.

Characteristics at baseline	Among the total population (N=149 444)		Among a sub-group not on disability pension at beginning of follow-up (N=139 220)	
	
	Reference group without mental disorders (N=124 536)	Individuals with ADHD, ASD or LD (N=24 908)	Reference group without mental disorders (N=123 227)	Individuals with ADHD, ASD or LD (N=15 993)
			
	RR (95% CI) ^a^	RR (95% CI) ^a^	RR (95% CI) ^a^	RR (95% CI) ^a^
Age at cohort entry (years)				
≤15	1.00	1.00	1.00	1.00
>15	1.01 (0.91–1.13)	1.03 (0.95–1.13)	0.98 (0.87–1.10)	1.46 (1.23–1.74)
Sex				
Men	1.00	1.00	1.00	1.00
Women	1.35 (1.22–1.49)	1.16 (1.08–1.25)	1.36 (1.23–1.52)	1.31 (1.12–1.53)
Educational level				
High	1.00	1.00	1.00	1.00
Intermediate	2.35 (1.98–2.79)	1.20 (0.98–1.47)	2.16 (1.80–2.59)	1.17 (0.81–1.68)
Low	18.40 (15.04–22.52)	2.98 (2.43–3.65)	4.02 (3.25–4.98)	2.10 (1.44–3.07)
Type of living area				
Large city	1.00	1.00	1.00	1.00
Medium-sized town	1.22 (1.10–1.36)	1.03 (0.94–1.12)	1.10 (0.98–1.23)	1.15 (0.97–1.36)
Small town/village	1.38 (1.23–1.54)	1.08 (0.99–1.18)	1.31 (1.17–1.48)	1.22 (1.02–1.46)
Birth country				
Sweden	1.00	1.00	1.00	1.00
Other	0.73 (0.61–0.88)	1.03 (0.89–1.18)	0.70 (0.58–0.85)	0.96 (0.71–1.29)

a Adjusted for age, sex, educational level, type of living area, birth country, and the year when follow-up began.

Table 5 shows the annual difference between diagnostics groups and the reference group. Although the overall pattern was similar to the original analyses, the differences seemed to slightly increase among men and slightly decrease among women.

**Table 5:** Adjusted rate ratio (RR) and 95% confidence intervals (CI) of the annual sickness absence and disability pension days among young adults diagnosed with attention-deficit/hyperactivity disorder (ADHD), autism spectrum disorder (ASD) or learning disabilities (LD) compared to a matched reference group without recorded mental disorders. The analyses are based on persons with full year follow-up periods.

	Reference group	ADHD	ASD	LD
		
		RR (95% CI) ^a^	RR (95% CI) ^a^	RR (95% CI) ^a^
Men				
Year 1	1.00	11.91 (10.31–13.75)	36.21 (30.05–43.65)	10.10 (5.78–17.64)
Year 2	1.00	12.30 (10.61–14.25)	39.24 (32.41–47.51)	10.65 (6.02–18.84)
Year 3	1.00	12.88 (11.00–15.07)	39.96 (32.75–48.74)	11.97 (6.48–22.12)
Women				
Year 1	1.00	13.58 (11.18–16.48)	31.41 (24.26–40.67)	11.21 (5.94–21.18)
Year 2	1.00	12.50 (10.30–15.17)	29.76 (23.02–38.48)	9.05 (4.82–16.97)
Year 3	1.00	12.04 (9.83–14.76)	28.79 (22.07–37.58)	8.77 (4.45–17.32)

a Adjusted for age, educational level, type of living area, birth country and year when follow-up began.

## Discussion

In this register-based prospective study of the working-age population in Sweden, we found high risk in terms of SA-DP associated with ADHD, ASD and LD. Two in three of the patients were treated in the specialized healthcare during childhood or adolescence, which reflects the early onset of these disorders. Compared to the matched reference group without recorded mental disorders, ASD was associated with the greatest burden of SA-DP with a 36-fold risk among men and 28-fold risk among women. Of them, 63% were already on DP at the beginning of follow-up. The corresponding risks among men and women with ADHD were 11 and 12; and among men and women with LD, 9 and 10, respectively. However, comorbid mental disorders played a significant role, further increasing the SA-DP risk. Educational disparities in SA-DP were smaller in the patient than reference group.

Our findings suggest that ASD is associated with the greatest burden of SA-DP. ASD represent a category of disorders that are characterized by difficulties in social reciprocity, communication and unusual or repetitive behavior. Among high-functioning people with ASD, difficulties in communication and social interaction rather than their actual work performance have been suggested to be the greatest obstacles to employment (4). Incapacity for work reflects a mismatch between job demands and an individual’s capacity to respond to these demands due to a physical disease or mental disorder. However, people with ASD often have special, although narrow skills and high competence, and it may be possible to support their employment and capacity for work. They may need specific support at the workplace; eg, specific time devoted to communication and a structured, non-complex work environment (4).

Our findings correspond with previous studies that have shown associations between ADHD and self-reported one-month SA days (15, 19), and short-term disability days (16). ADHD is associated with symptoms that may substantially reduce work performance, such as abnormal attention, impulsiveness, hyperactivity or restlessness, disorganization, and time management and memory problems (14). People with ADHD may also have problems in motor coordination as well as in working memory, planning and anticipation, verbal fluency, effort allocation, and self-regulation of emotional arousal. However, they often have a high level of energy and enthusiasm, which may be a great advantage in favorable circumstances. Therefore, it has been recommended that regarding employees with ADHD, special attention should be paid to calming the work environment (eg, by avoiding noise and open offices). They should be allowed to move during the workday and receive help in organizing and keeping time schedules, and planning work tasks. They should be able to delegate work and receive support from supervisors and co-workers (14). Early ADHD has been found to be associated with later mental and substance use disorders, early pregnancy, school drop-out and criminality (5), which provides an idea of the potential mechanism linking early ADHD with future incapacity for work. In addition, behavior-based treatments may be effective, in addition to stimulant medication treatment (21).

LD refers to a heterogeneous group of neurodevelopmental disorders that influence the individual’s ability to maintain, process or convey information to others effectively (22). This is often comorbid with other neurodevelopmental disorders such as ADHD (2). The literature on adult outcomes of LD is limited, and focuses mainly on educational and employment outcomes, showing mixed findings. The authors of a recent systematic review (2) concluded that comorbid mental disorders are the primary drivers of unfavorable outcomes in LD and a source of heterogeneity among previous studies. Our findings give some support to this hypothesis although the associations of comorbidity were not statistically significant.

Of the socioeconomic factors, educational disparities in SA-DP were smaller in the patient groups than the reference group without recorded mental disorders. It is possible that having a neurodevelopmental disorder per se is highly disabling and therefore less affected by social or environmental factors. There is some evidence from the US that neurodevelopmental disorders, such as ASD are underdiagnosed among socioeconomically disadvantaged groups due to worse resources and poorer access to health services compared to more affluent groups (23). If this was the case also in Sweden, proportionally more undiagnosed cases with socioeconomic disadvantage would have been in our reference group. These issues need to be investigated in detail in future studies. In general, the reference group consisted of young people who were not treated in specialized mental healthcare but may have had treatment contact in primary healthcare.

The specific strength of this study is its design based on nationwide registers that cover the entire Swedish population, and the high coverage and good validity of the Swedish health registers (24). The large database enabled us to investigate young adults with relatively rare neurodevelopmental disorders. The associations were quite similar in sub-group analyses excluding those with DP at baseline, although the absolute rates were lower, as expected.

However, the number of individuals with LD was lower than would be expected from prevalence statistics; the individuals with LD in our study were probably severe enough to be treated in a specialized healthcare setting. Furthermore, some individuals may have had their diagnosis before age 10 and not been in contact with specialized healthcare after that. However, as the registers cover both specialized outpatient clinics and inpatient healthcare, we do not consider this a major source of misclassification. Regarding comorbidity, some milder comorbid disorders, such as milder depressive disorders, may have had been misclassified because they are often treated in primary healthcare. The follow-up in our study was three years which reflects relatively short-term impacts of neuropsychiatric diseases in adult life. Work incapacity usually increases with age, thus future studies with longer follow-up may observe greater increases. The MIDAS register of SA-DP is valid in terms of high coverage and being based on a physician-assessed diagnosis of a disease and disability. One limitation is that our findings may be generalizable to cases treated in specialized healthcare and may not be generalizable to countries with very different social and healthcare systems. Finally, there are other risk and protective factors that were not assessed in the present study.

In conclusion, our findings suggest that ADHD, ASD, and LD have major effects on the lives of young adults in terms of incapacity for work. The greatest burden was found to be associated with ASD. Specific attention should be paid to co-occurring mental disorders that considerably impair the prognosis. Efforts to improve employment in these groups may include, for example, the individual placement and support (IPS) method which has been shown to be effective among young adults (25).
